# Overexpression of CD90 (Thy-1) in Pancreatic Adenocarcinoma Present in the Tumor Microenvironment

**DOI:** 10.1371/journal.pone.0115507

**Published:** 2014-12-23

**Authors:** Jianhui Zhu, Smathorn Thakolwiboon, Xinhua Liu, Min Zhang, David M. Lubman

**Affiliations:** 1 Department of Surgery, University of Michigan Medical Center, Ann Arbor, Michigan 48109, United States of America; 2 Department of Medicine, Faculty of Medicine Siriraj Hospital, Mahidol University, Bangkok 10700, Thailand; 3 Experiment Center for Science and Technology, Shanghai University of Traditional Chinese Medicine, Shanghai 201203, China; 4 Department of Biostatistics, University of Michigan School of Public Health, Ann Arbor, Michigan 48109, United States of America; Garvan Institute of Medical Research, Australia

## Abstract

CD90 (Thy-1) plays important roles in oncogenesis and shows potential as a candidate marker for cancer stem cells (CSCs) in various malignancies. Herein, we investigated the expression of CD90 in pancreatic adenocarcinoma (PDAC), with a comparison to normal pancreas and non-malignant pancreatic disease, by immunohistochemical (IHC) analysis of tissue microarrays containing 183 clinical tissue specimens. Statistical analysis was performed to evaluate the correlation between CD90 expression and the major clinicopathological factors after adjustment of age and gender. The IHC data showed that CD90 was significantly overexpressed in PDAC and its metastatic cancers as compared to chronic pancreatitis and benign islet tumors, while it was negative in normal pancreas and 82.7% of adjacent normal pancreas tissues. The abundant CD90 expression was predominantly present in PDAC stroma, such as fibroblasts and vascular endothelial cells, which could serve as a promising marker to distinguish pancreatic adenocarcinoma from normal pancreas and non-malignant pancreatic diseases. Double immunostaining of CD90 with CD24, a CSC marker for PDAC, showed that there was little overlap between these two markers. However, CD90^+^ fibroblast cells were clustered around CD24^+^ malignant ducts, suggesting that CD90 may be involved in the tumor-stroma interactions and promote pancreatic cancer development. Furthermore, CD90 mostly overlapped with α-smooth muscle actin (αSMA, a marker of activated pancreatic stellate cells (PSCs)) in PDAC stroma, which demonstrated that CD90^+^ stromal cells consist largely of activated PSCs. Double immunostaining of CD90 and a vascular endothelial cell marker CD31 demonstrated that CD90 expression on vascular endothelial cells was significantly increased in PDACs as compared to normal pancreas and non-malignant pancreatic diseases. Our findings suggest that CD90 could serve as a promising marker for pancreatic adenocarcinoma where desmoplastic stroma plays an important role in tumor growth and angiogenesis.

## Introduction

CD90 (Thy-1) is a 25–37 kDa glycosylphosphatidylinositol (GPI)-anchored glycoprotein expressed on many cell types [1]. In humans, CD90 is expressed on fibroblasts, neurons, blood stem cells and activated microvascular endothelial cells (ECs) [2], [3]. CD90 has been used as a cell marker for isolation of stem cells, such as bone marrow-derived mesenchymal stem cells [4] and hepatic stem/progenitor cells (HSPCs) [5], [6]. It is an important regulator of cell-cell and cell-matrix interactions, with significant roles in cellular adhesion and migration, nerve regeneration, and fibrosis [7].

CD90 also plays important roles in oncogenesis and shows potential as a candidate marker for cancer stem cells (CSCs) in various malignancies, such as hepatocellular carcinoma (HCC) [8], esophageal cancer [9], and glioma [10]. The CD90^+^ cells enriched from human HCC cell lines, but not the CD90^−^ counterpart, displayed tumorigenic capacity [8]. In contrast, the CD90^+^CD45^−^ population was detected in all liver tumor specimens and 91.6% of blood samples of liver cancer patients, which could generate tumor nodules in immunodeficient mice [8]. The CD90^+^ cell population isolated from esophageal squamous cell carcinoma (ESCC) was also found to possess stem cell-like properties and highly tumorigenic and metastatic potential [9]. CD90 was identified in both primarily cultured CD133^+^ glioma CSCs [11] and glioma tissue specimens, exhibiting a medium to high level in high-grade gliomas [10]. It was predominantly co-localized with CD31, a vascular endothelial cell marker, which indicated that CD90^+^ glioma CSCs may reside within the endothelial niche for their self-renewal [10]. However, little is known about the expression pattern of CD90 in pancreatic adenocarcinoma and its potential role in the tumorigenesis of PDAC.

In this study, CD90 was investigated to characterize its potential in PDAC by immunohistochemical (IHC) analysis of tissue microarrays which contain 183 clinical tissue specimens. Statistical analysis was performed to evaluate the correlation between CD90 expression and the major clinicopathological factors after adjustment of age and gender. It is shown that the expression level of CD90 is significantly increased in PDAC patients as compared to normal pancreas, adjacent normal tissue, chronic pancreatitis and benign islet tumors. In PDACs, the expression of CD90 was abundantly present in the stroma, such as fibroblasts and vascular endothelial cells. Double immunofluorescence staining of CD90 and CD24 showed that the abundant CD90^+^ fibroblasts were clustered around CD24^+^ malignant ducts, suggesting that CD90 may be involved in the tumor-stroma interactions and promote pancreatic cancer development. Double staining of CD90 and αSMA (a PSC activation marker) showed that CD90 mostly overlapped with αSMA in PDAC stroma, indicating that CD90^+^ stromal cells consist largely of activated PSCs which contribute to fibroblastic proliferation and fibrosis in PDAC. Furthermore, CD90 expression on vascular endothelial cells was significantly elevated in PDACs compared to normal pancreas and non-malignant pancreatic diseases. These results suggest that CD90 may play a potential role in the promotion of tumor growth and angiogenesis in pancreatic adenocarcinoma.

## Materials and Methods

### Tissue Specimens

The tissue microarrays (TMAs) of formalin-fixed paraffin-embedded pancreatic tumors and normal tissues were purchased from US Biomax Inc. (Rockville, MD). Tissue specimens comprised 20 normal human pancreas (age: from 21 to 50 years, median: 35 years) and 163 cases of patients (age: from 31 to 78 years, median: 56 years), including pancreatic adenocarcinoma (n = 98) with clinical stages and pathology grades, pancreatitis (n = 11), metastasis (n = 6), benign islet cell tumor (n = 10), islet cell carcinoma (n = 4), pancreatic adenosquamous carcinoma (n = 5), and cancer adjacent normal pancreas (n = 29). The tissue specimens originated from different donors and two replicate biopsies of each patient were spotted on the same block and were analyzed *in situ*. The IHC staining showed consistent results between the 2 replicates. The clinical characteristics of patients are listed in Table 1.

**Table 1:** Clinical pathologic characteristics of the patient samples (n = 163) in TMAs.

Median age	56 y (range, 23–78 y)
**Gender (male/female)**	93/70
**Pancreatic Adenocarcinoma (n = 98)**	
**TNM Stage**	
I/II/III/IV	42/42/9/5
**Pathological** **grade**	
G1/G2/G3	23/33/35
Not assessed	7
**Pancreatitis**	11
**Metastasis**	6
**Islet cell tumor (Benign)**	10
**Neuroendocrine carcinoma**	4
**Adenosquamous carcinoma**	5
**Adjacent Normal Tissue (ANT)**	29

### Immunohistochemistry of CD90

Immunohistochemical staining was performed on tissue microarrays. The FFPE tissue arrays were dewaxed in xylene for 10 min and rehydrated through a series of alcohol solutions (100% ethanol twice, 95% ethanol, 75% ethanol, 5 min each) to water. Antigen retrieval was achieved by boiling the arrays in a citrate buffer at pH 6.0 (Invitrogen, Grand Island, NY). The arrays were then examined by immunoperoxidase and immunofluorescence methods, respectively.

In the immunoperoxidase method, an extra step was taken to block endogenous peroxidase activity by using 6% H_2_O_2_. Subsequently, the arrays were treated with 2% BSA in PBST for 1 hr to block non-specific binding and then incubated with rabbit anti-human CD90 monoclonal antibody (1∶100 dilution, Abcam, Cambridge, MA) overnight at 4°C. The arrays were then stained with horseradish peroxidase (HRP)-conjugated anti-rabbit IgG antibody (Abcam, Cambridge, MA) for 1 hr at room temperature and developed in 3,3′-diaminobenzidine (DAB) solution (Vector Labs, Burlingame, CA) according to the manufacturer's instructions. The immunoreactivity is shown in *brown*. Hematoxylin counterstain was applied to visualize nuclei (*blue*). Between each step, there were three washes with PBST for 10 min each. Finally, the TMAs were dehydrated in gradient alcohol and xylene, and then coverslipped using a CC/Mount permanent mounting medium (Sigma).

In the immunofluorescence (IF) method, the DyLight 488 conjugated anti-rabbit IgG antibody (Vector laboratories, Burlingame, CA) was applied as a secondary antibody and incubated with the arrays at a dilution of 1∶200 for 1 hr at room temperature. CD90 staining is shown in *green*. Nuclei visualization was explored by DAPI counterstaining (*blue*).

The isotype control was conducted to eliminate non-specific interactions between IgG molecules of the rabbit anti-CD90 antibody and tissue samples. The sample was incubated with a rabbit IgG monoclonal isotype control (1∶100 dilution, Abcam, Cambridge, MA), in place of the primary CD90 antibody, followed by incubation with the secondary antibody and detection reagents as described above.

### Double Immunofluoresence Staining of CD90 with Known Markers

Subclassification of the CD90^+^ population was investigated by the double immunostaining of CD90 with CD24 (CSC marker for PDAC), and cellular markers including α-smooth muscle actin (αSMA, a marker of activated pancreatic stellate cells), CD31 (endothelial vascular cell marker) and CD45 (leukocyte common antigen), respectively. The TMAs were dewaxed, rehydrated, and treated with citrate buffer for antigen retrieval and then 2% BSA to block non-specific binding as described above. To achieve double immunofluorescence (IF) staining of CD90 and the known markers, rabbit anti-CD90 (Abcam, Cambridge, MA) was mixed with mouse anti-CD24 (Abcam, Cambridge, MA), mouse anti-αSMA (Sigma), mouse anti-CD31 (Novocastra, Newcastle Upon Tyne, UK), and mouse anti-CD45 (Abcam, Cambridge, MA) antibodies, respectively, at the optimal dilutions according to the manufacturer's instructions. The antibody mixture was then incubated with the TMAs overnight at 4°C. Then DyLight 549 anti-mouse IgG (*red*) and DyLight 488 anti-rabbit IgG (*green*) (Vector laboratories, Burlingame, CA) were diluted (1∶200) and incubated with the TMAs for 1 hr at room temperature. Nuclei visualization was explored by DAPI counterstaining (*blue*). The TMAs were finally dehydrated in alcohol and coverslipped.

### Evaluation of Immunohistochemical Staining

IHC score was based upon the product of the percentage CD90^+^ cells multiplied by stain intensity (0 =  negative, 1 =  weak, 2 =  moderate, 3 =  strong) for each specimen. The percentage of CD90^+^ cells (%pos; 0 =  no staining, 1 = <10%, 2 = 10–50%, 3 = 51–80%, 4 = >80%) was assessed by the mean of %pos in three different microscopic fields under 200× magnification on a Nikon Eclipse T*i* microscope accompanied by imaging software NIS-Elements AR 4.13.00. When the multiplication product (from 0 to 12) of the percentage of positive cells and stain intensity was ≥2, the specimen was considered positive for CD90 expression as described previously [12].

### Statistical Analysis

The association between CD90 expression and disease types was studied using the analysis of covariance (ANCOVA), adjusted for patient age and gender. Pair-wise comparison of IHC scores between each group, including normal pancreas, adjacent normal tissue, pancreatitis, benign islet cell tumor, PDAC of stage I, II, and III/IV, and metastasis, was made by comparing the least square means and the multiple comparisons were adjusted by the Tukey's method. Further, linear regression analysis was used to assess the association of clinicopathological variables with IHC scores. A value of *p*<0.05 was considered statistically significant. The scatter plot of the IHC scores among groups was generated with GraphPad Prism 6 (La Jolla, CA), with reference lines at the mean expression, which allows for an assessment of the correlation between CD90 expression and tumor progression. The receiver operating characteristics (ROC) curve of CD90 expression between the study groups was analyzed using Prism 6 (La Jolla, CA).

## Results

First, we evaluated the specificity of the rabbit anti-human CD90 antibody (Abcam, Cambridge, MA). The isotype control showed that there was no background staining for the rabbit IgG on pancreatic tissue arrays in both immunoperoxidase and immunofluorescence methods, while CD90 showed abundant staining (Figure S1 in S1 File). This demonstrated that the specific staining for CD90 was not caused by non-specific interactions of IgG molecules with the tissue samples. The specificity of the CD90 antibody was also evaluated by western blotting as shown on the vendor website (http://www.abcam.com/cd90-thy1-antibody-epr3132-ab92574.html), with a single band at ∼25 kDa in human tissue lysates. These observations confirmed that the rabbit anti-CD90 antibody specifically reacts to the antigen and is suitable for the immunostaining studies.

### CD90 Expression in Normal Pancreas

**Figure 1:**
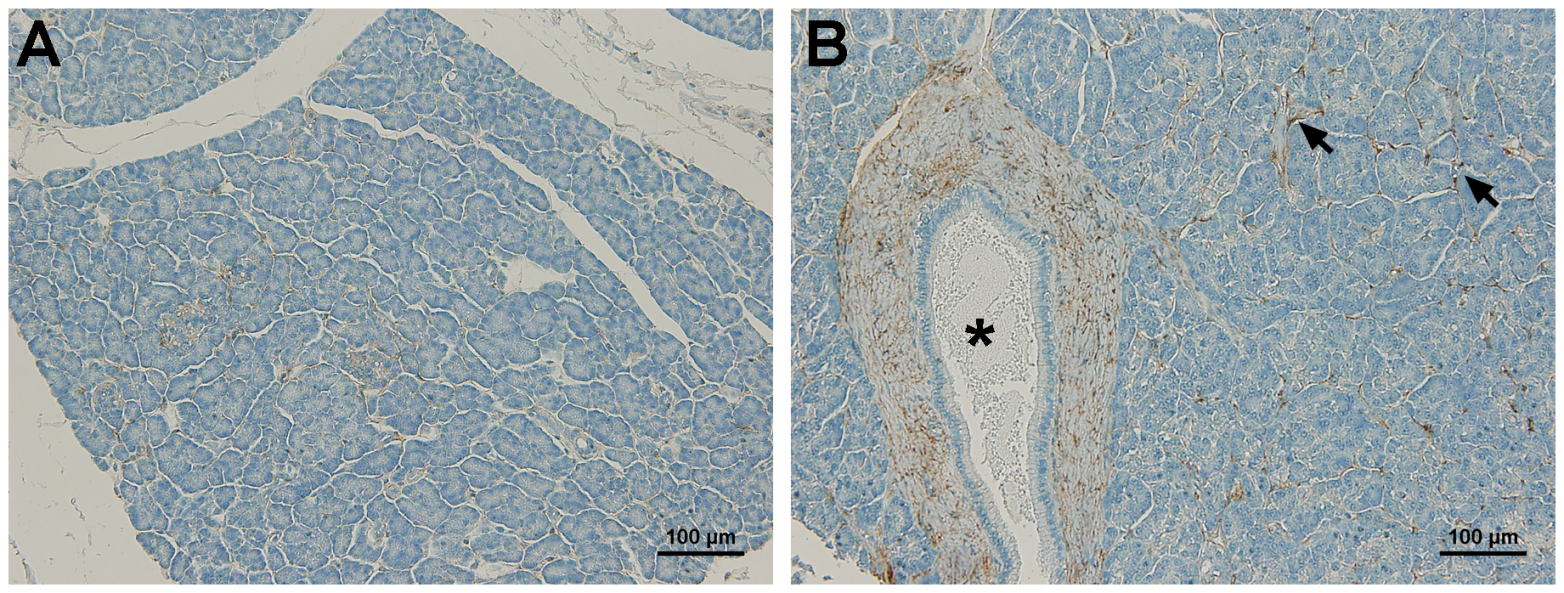
CD90 expression in normal human pancreas. (A) A representative image of normal pancreas showed negative expression of CD90. (B) Minimal CD90 expression was observed on the connective tissues (arrows). The fibroblasts surrounding the pancreatic main duct (asterisk) showed weak CD90 expression. No expression of CD90 was observed on ducts, acini, islets, and blood vessels. Scale bars  = 100 µm.

In normal pancreas, CD90 showed weak and sparse staining on the connective tissues, with less than 1% CD90^+^ cells, which was considered as negative. A representative image of normal pancreas with negative CD90 expression is shown in Fig. 1A. Fig. 1B showed weak CD90 expression (light *brown*) on the connective tissues and fibroblasts around the main pancreatic duct (asterisk). Note that CD90 staining was restricted on the connective tissues while no expression was observed in pancreatic ducts, acini, islets, or blood vessels.

We also examined CD90 expression on the histologically normal tissue adjacent to pancreatic adenocarcinoma. 82.7% (24/29) of adjacent normal tissues were negative for CD90. As shown in Figure S2 in S1 File, minimal CD90 expression was observed in the connective tissues. The fibroblasts that are located adjacent to ducts showed increased expression of CD90 (Figure S2 in File S1). In adjacent normal tissues, there was also no expression observed in the pancreatic ducts, acini, islets, or blood vessels.

### CD90 Expression in Non-malignant Pancreatic Disease

**Figure 2:**
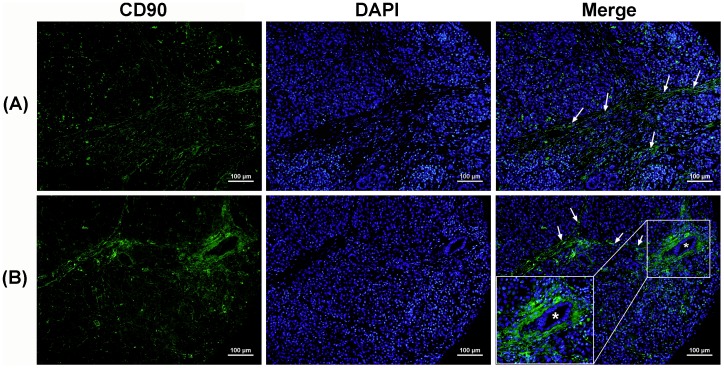
Increased CD90 expression (*green*) was observed on the activated fibroblasts in chronic pancreatitis. (A) A representative image of pancreatitis tissue showed moderate expression of CD90 in fibroblast cells (indicated by arrows). (B) Fibroblasts around a duct (asterisk) showed distinctly strong expression of CD90 in pancreatitis. The insert is a higher magnification of the ductal area. Nuclei were stained with DAPI (blue). Scale bars  = 100 µm.

Next, we investigated CD90 expression in non-malignant pancreatic disease, including chronic pancreatitis and benign islet cell tumors. In chronic pancreatitis, which is characterized by ongoing inflammation of the pancreas, the immunofluorescence staining showed that an increased expression of CD90 was observed on the activated fibroblasts. CD90 expression was found in 36% (4/11) of chronic pancreatitis. As shown in Fig. 2, a moderate to strong CD90 expression (*green*) was observed in fibroblasts (indicated by arrows). The insert in Fig. 2B is a high magnification of a pancreatic duct around which distinctly strong CD90 expression was observed on the fibroblasts. There was no CD90 expression observed in the acinar and ductal epithelia, or blood vessels in chronic pancreatitis.

The islet cell tumor, also called pancreatic neuroendocrine tumor, is a mass of abnormal cells that forms in the endocrine tissues of pancreas, which can be benign or malignant. In benign islet cell tumors, 80% (8/10) of cases were negative for CD90. Only 20% of benign islet cell tumors showed moderate CD90 expression, which was present on both fibroblasts and tumor cells (Figure S3 in S1 File).

### Overexpression of CD90 in Pancreatic Cancer

**Figure 3:**
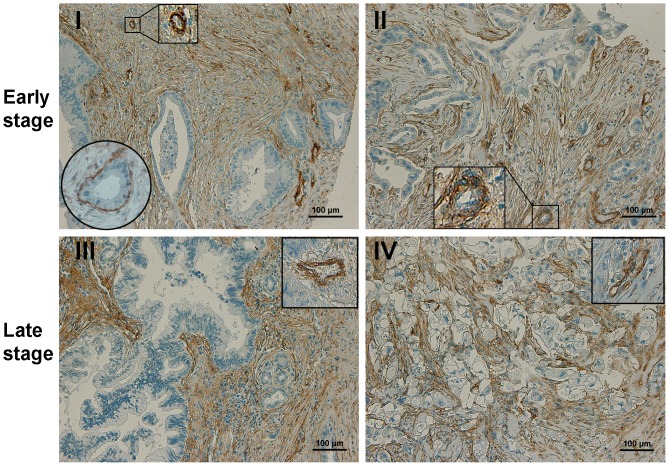
Overexpression of CD90 in the stroma of pancreatic adenocarcinoma (PDAC). In both early (I and II) and late stages (III and IV) of PDACs, CD90 expression was highly increased, which was abundantly present in the activated stroma, including fibroblasts and vascular endothelial cells. The positive CD90^+^ stromal cells were clustered around the malignant ducts. The circle insert in 3I showed a single layer of CD90^+^ fibroblasts closely localized around tumor duct. The square inserts in 3I–3IV showed strong CD90 expression on vascular endothelial cells in PDAC stage I, II, III and IV, respectively.

Pancreatic adenocarcinoma (PDAC) is the most common type of pancreatic malignancies, accounting for over 90% of all pancreatic cancers. In PDACs, CD90 was highly overexpressed. 92.8% (91/98) of PDAC specimens were positive for CD90, with strong or moderate staining on stromal cells. As shown in Fig. 3, CD90 was abundantly expressed in the activated stromal cells, including fibroblasts and vascular endothelial cells, in both early and late stages of PDAC. Abundant CD90^+^ fibroblasts were present in all stages, which were found around/related to malignant ducts, as well as blood vessels and pancreatic lobules. Note that, in stage I, a single layer of CD90^+^ stromal fibroblastic cells was surrounding the duct-like structure (marked by a circle in Fig. 3I). In addition, CD90 expression on vascular endothelial cells was significantly increased in all stages of PDAC (square inserts in Fig. 3I-3IV), which could distinguish PDAC from normal and non-malignant pancreatic tissues.

**Figure 4:**
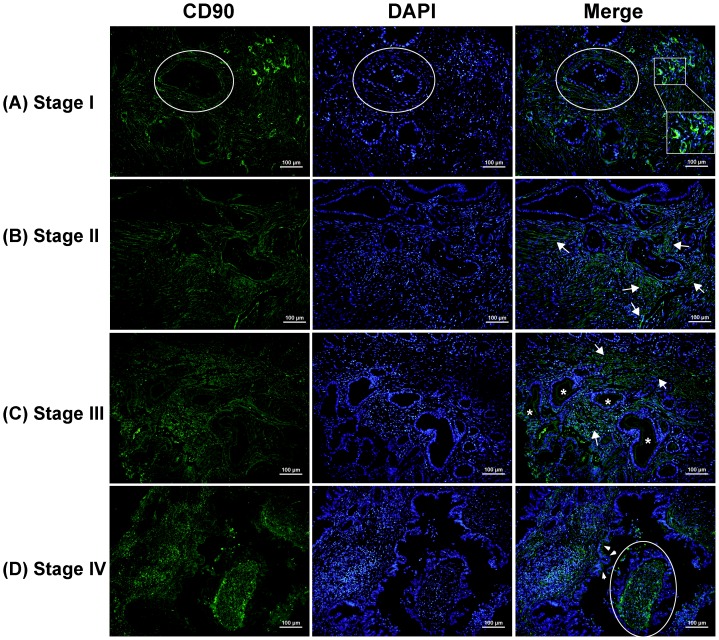
Distribution of CD90 in PDAC of early and late stages evaluated by immunofluorescence staining (*green*). DAPI counterstaining was used to visualize nuclei (*blue*). In early-stage PDACs, CD90 is abundantly expressed on stromal cells, including activated fibroblasts and vascular cells which form the basis for blood vessels (shown in square). Membrane expression of CD90 on the apical cell surface was rarely observed in malignant ducts in late-stage PDACs (marked with a star or arrow). Expression was also observed on endothelial cells (in red circle). Scale bars  = 100 µm.

We further evaluated the subcellular distribution of CD90 expression in PDACs by using immunofluorescence staining (Fig. 4). CD90 expression was dominantly present in fibroblasts. The insert in Fig. 4I and 4III showed membrane expression of CD90 on vascular endothelial cells. It is notable that, in late stages of PDAC, membrane expression of CD90 was rarely observed on the apical cell surface of malignant ducts which were marked with asterisks in Fig. 4III and indicated with arrows in Fig. 4IV. The detection of CD90 expression on tumor cells in late-stage PDAC in the immunofluorescence staining method may be because the fluorophore is several times more sensitive than the chromophore, such as DAB used in the immunoperoxidase method. Abundant cytoplasmic expression was also observed on endothelial cells in late stage PDACs (Fig. 4IV).

The expression of CD90 in metastatic PDAC that has spread to liver, lymph node, abdominal cavity, or omentum, was also investigated (Figure S4 in S1 File). As shown in Figure S4 in S1 File, moderate to strong expression of CD90 was observed on fibroblasts in these metastatic carcinomas. In addition, the metastatic carcinoma to abdominal cavity also showed strong CD90 expression on the perivascular niche, suggesting the angiogenesis potential of CD90^+^ cells in metastatic cancer.

In addition to PDAC, we investigated the expression of CD90 in malignant pancreatic neuroendocrine tumor (PNET) and adenosquamous carcinoma (PASC), which are two rare subtypes of pancreatic cancer accounting for less than 5% [13] and 4% [14] of all pancreatic malignancies, respectively. Due to the limited access to PNET and PASC samples in the United States, only 4 cases of PNET (also called islet cell carcinoma) and 5 cases of PASC tissue samples were included in this study. As shown in Figure S5 in S1 File, strong CD90 expression (*green*) was present on the endothelial tumor cells in PNET samples. However, in PASC, strong CD90 expression was observed in both stromal cells (indicated by arrows) and tumor cells (Figure S5 in S1 File). The insert in Figure S5 in S1 File is a higher magnification showing CD90 expression on tumor cells in PASC. Due to the small sample size of PNET and PASC patients, the further statistical analysis of CD90 expression in pancreatic cancer was focused on patients with PDAC.

### Correlation of CD90 Expression with Clinicopathological Variables

To assess the association between CD90 expression and disease types, we first tested whether the distribution of patient characteristics, such as age and gender, are similar across all disease types analyzed in this study. The ANOVA analysis showed that the normal pancreas group was on average younger than cancer groups (*p*<0.001), which is mainly because pancreatic cancer incidence is strongly related to age. In addition, the normal group had more male subjects compared to other groups and the Chi-square test suggested a significant difference in gender across disease types (*p* = 0.05). Therefore, statistical analyses of the association between CD90 expression and disease types were adjusted for age and gender using linear regression, also commonly referred to as ANCOVA in this setting.

**Figure 5:**
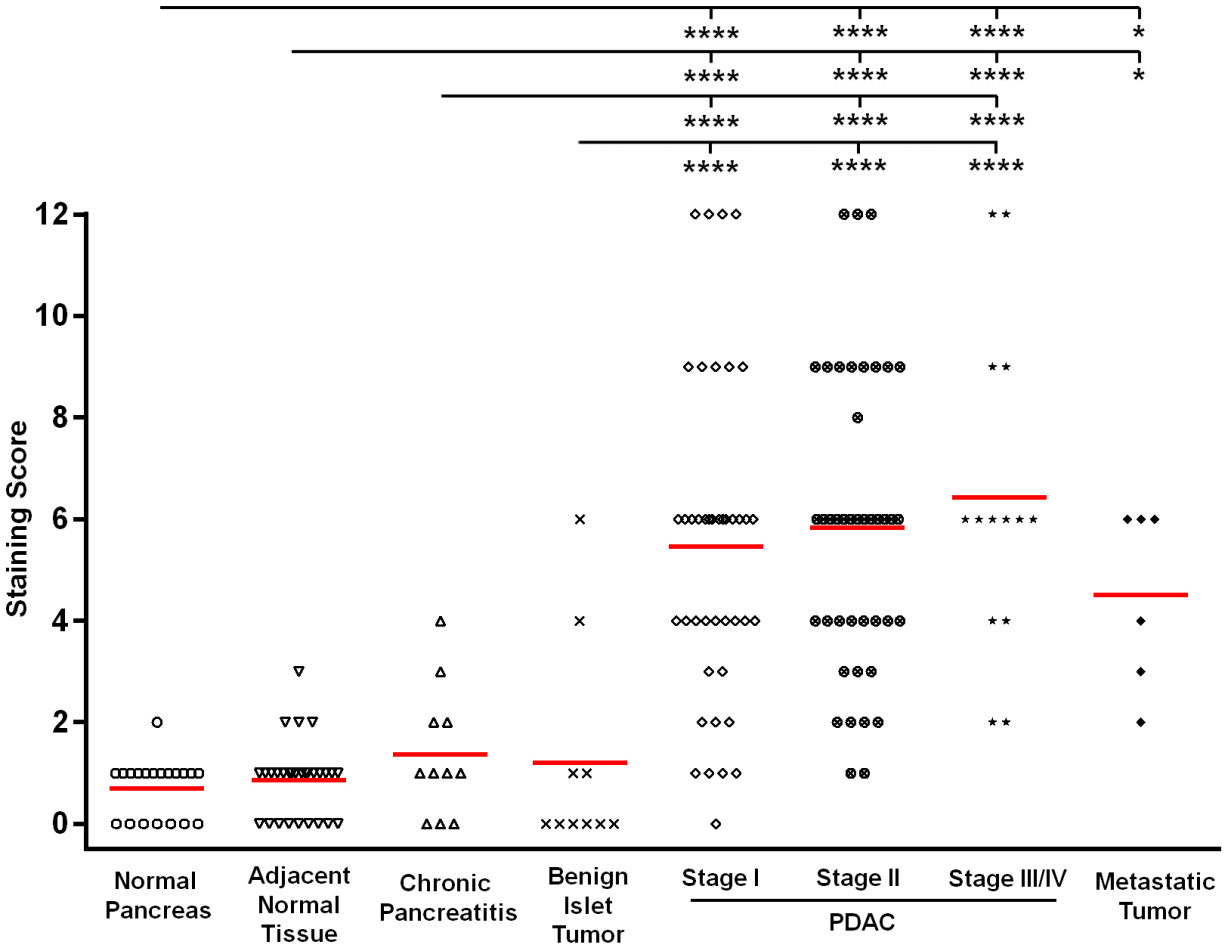
Comparison of staining score of CD90 expression among normal pancreas, cancer adjacent normal tissue, chronic pancreatitis, benign islet tumor, PDAC of stage I, II, and III/IV, and metastatic tumor. *P*-values were determined in ANCOVA by comparing IHC scores between each group, adjusted for age and gender, and the multiple comparisons are accounted for using the Tukey's method. The CD90 staining score was distinctly higher in PDAC of all stages compared to those in non-malignant cases (*p*<0.0001). Samples with metastatic tumors also showed increased expression of CD90 than in non-malignant cases (*p*<0.05). (*: *p*<0.05; ****: *p*<0.0001).

CD90 expression in different patient groups was evaluated by the IHC staining score (from 0 to 12), which was based on the product of percentage CD90^+^ cells (%pos; 0  =  no staining, 1 = <10%, 2 = 10–50%, 3 = 51–80%, 4 = >80%) multiplied by stain intensity (0 =  negative, 1 =  weak, 2 =  moderate, 3 =  strong). As shown in Fig. 5, the CD90 staining score (mean (SEM)) in normal pancreas, adjacent normal tissue, chronic pancreatitis, and benign islet tumor was extremely low, which was 0.70 (0.13), 0.80 (0.14), 1.36 (0.39), and 1.20 (0.66), respectively. There was no significant difference in CD90 expression between non-malignant cases and normal pancreas group. However, the staining score of CD90 was distinctly increased in PDAC of all stages compared to that in non-malignant cases (*p*<0.0001). The staining score (mean (SEM)) of CD90 in PDAC stage I, II, III/IV, and metastasis was 5.45 (0.49), 5.83 (0.45), 6.43 (0.84), and 4.50 (0.72), respectively. Samples with metastatic pancreatic cancer also showed significantly increased expression of CD90 compared to non-malignant cases (*p*<0.05). In addition to the raw group-specific means shown above, the age and gender adjusted least square mean for each group is shown in Table 2. The overall test for testing the association of disease type with CD90 expression yields a *p*-value less than 0.0001, indicating that CD90 expression is strongly associated with disease type. Further, the receiver operating characteristics (ROC) analysis indicated that CD90 expression could distinguish PDAC from normal, chronic pancreatitis and benign islet tumors with a specificity of 90% at a sensitivity of 84% (AUC = 0.937). These results suggest that CD90 may serve as a promising marker for pancreatic adenocarcinoma and CD90^+^ cells might be associated with distant metastasis.

**Table 2:** Relationship between CD90 expression and pancreatic disease types using linear regression model after adjustment for age and gender.

Characteristic	Coefficient/LS mean (SE)	*p*-value
**Age**	0.004*^a^* (0.0018)	0.84
**Gender (female = 1, male = 0)**	−0.64*^a^* (0.40)	0.10
**Disease type**		<0.0001^c^
**Normal**	0.62^b^ (0.63)	
**Adjacent normal Tissue**	0.85^b^ (0.46)	
**Benign islet tumor**	1.43^b^ (0.79)	
**Chronic pancreatitis**	1.41^b^ (0.74)	
**PDAC**	5.76^b^ (0.25)	
**Metastasis**	4.35^b^ (1.00)	

Note: *^a^*indicates the coefficient.

^b^represents the least squares mean (LS mean) adjusted for age and gender.

^c^The overall significance of CD90 expression among disease types.

We also assessed the correlation of CD90 expression with the major clinicopathological factors among PDAC subjects and results are shown in Table 3. The result showed that CD90 expression was not associated with age (*p* = 0.63) and no significant difference of CD90 staining score was detected among various PDAC stages (*p* = 0.39), probably due to small sample size. It was found that gender (*p* = 0.03) and PDAC grade (*p* = 0.009) are associated with CD90 expression, with females showing less CD90 expression and CD90 expression decreasing with grades.

**Table 3:** Relationship between CD90 expression and clinicopahological factors among patients with pancreatic adenocarcinoma (PDAC).

Characteristic	Coefficient/LS Mean (SE)	*p*-value
**Age**	0.014*^a^* (0.028)	0.63
**Gender (female = 1, male = 0)**	−1.40*^a^* (0.63)	0.03
**Grade**		0.009
Grade 1	7.19^b^ (0.61)	
Grade 2	6.24^b^ (0.51)	
Grade 3	4.82^b^ (0.49)	
**Stage**		0.39
Stage I	5.37^b^ (0.47)	
Stage II	5.84^b^ (0.47)	
Stage III/IV	6.66^b^ (0.82)	

Note: *^a^*indicates the coefficient.

^b^represents the least squares mean (LS mean) adjusted for age and gender.

### Subcellular CD90 and CD24 Expression

**Figure 6:**
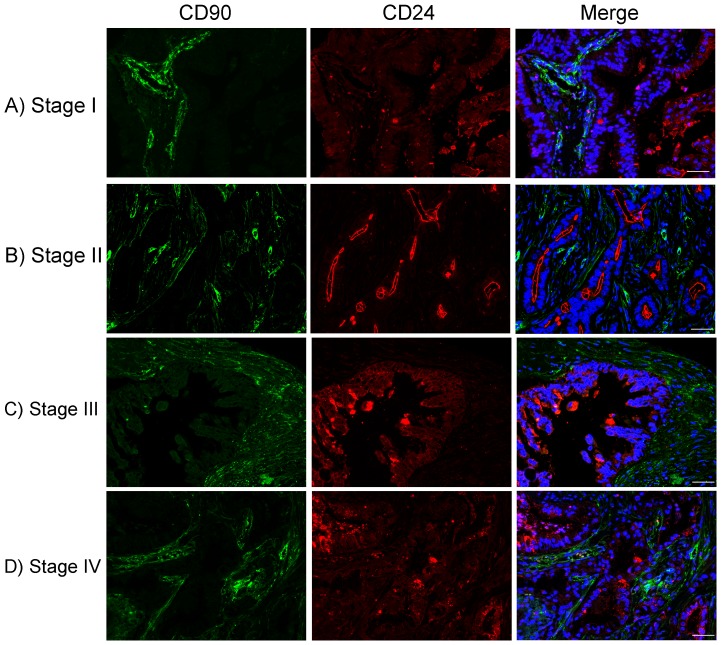
CD90 (*green*) and CD24 (*red*) expression in PDAC of early (A and B) and late (C and D) stages. DAPI counterstaining was used to visualize nuclei (*blue*). Scale bars  = 50 µm. There was little overlap between CD90 and CD24. However, CD90^+^ stromal fibroblasts were localized to tumor glands where CD24 shows strong expression on the cell surface membrane, suggesting that cancer-associated stroma has a function in tumor progression.

The double fluorescence IHC staining was performed to investigate the overlap between CD90 and CD24, one of CSC markers for PDAC, and their subcellular expression patterns. Consistent with a previous IHC study [15], CD24 showed sparse expression in acinar cells in normal pancreas tissues (data not shown), which was considered negative. In PDACs, CD24 expression was observed in 87% of cases, with strong or moderate staining on both membrane and cytoplasm of tumor cells (Fig. 6). CD24 exhibited a higher expression predominantly present on the apical membrane of malignant ducts and vessels in high-stage tumors (Fig. 6), as reported previously [16]. Compared to the dominant expression of CD24 on the membrane of malignant tumor cells, CD90 was mainly localized in the stroma, including fibroblasts and vascular endothelial cells. Although there was little overlap between CD90 and CD24, interestingly, CD90^+^ cells were clustered in three to five layers around CD24^+^ malignant ducts (Fig. 6B and 6C), suggesting that CD90 may play a critical role in cancer-associated fibroblasts which can promote pancreatic tumorigenesis and development.

### Coexpression of CD90 with αSMA

**Figure 7:**
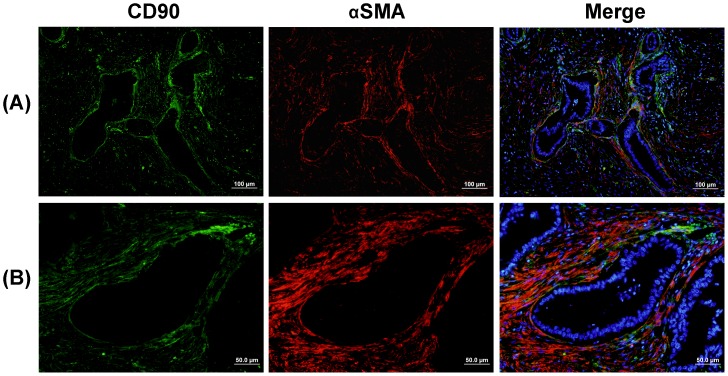
Double immunofluoresence staining of CD90 (*green*) and αSMA (*red*) on PDAC tissues. DAPI counterstaining was used to visualize nuclei (*blue*). *Yellow* or *orange* represent the co-localization of CD90 and αSMA. (A) A stage I PDAC specimen exhibited a single layer of CD90^+^ activated PSCs (*yellow*) surrounding the duct-like structure. (B) Higher magnification of the stromal area in PDAC displaying an apparent overlap between CD90 and αSMA (y*ellow* or *orange* in the merged images). The IHC result showed that CD90 mostly overlapped with αSMA in PDAC stroma, demonstrating that CD90^+^ stromal cells consist largely of activated PSCs.

Since CD90 expression was abundantly observed on fibroblasts in PDAC stromal areas while pancreatic stellate cells (PSCs) are a major contributor to fibroblastic proliferation and fibrosis in PDAC [17], we further performed double IF staining of CD90 and αSMA (a PSC activation marker) to investigate the CD90^+^ population in pancreatic stellate cells (PSCs). As shown in Fig. 7, the double IF staining revealed that CD90 mostly overlapped with αSMA (*yellow* or *orange* in the merged images) in PDAC stroma, which demonstrate that CD90^+^ stromal cells consist largely of activated PSCs. The result is also consistent with the fact that PSCs are a major contributor to fibroblastic proliferation and fibrosis in PDAC. Interestingly, in stage I, a single layer of CD90^+^ activated PSCs surrounded the duct-like structure (Fig. 7A), suggesting that CD90^+^ PSCs may play an important role in tumor-stroma interactions in PDAC and modulation of fibroblast proliferation.

Sirius Red staining has also been performed to evaluate the correlation of CD90 expression and the degree of collagen deposition. As shown in Figure S6 in S1 File, Sirius Red stained for collagen in stromal areas of PDAC, which also exhibited strong and abundant expression of CD90 (*green*) and αSMA (*red*). No collagen staining was observed in tumoral areas. Apte *et al.* has reported that PDAC patients showed colocalization of staining for collagen and αSMA in stromal areas, indicating that activated PSCs were the predominant source of collagen in the stromal reaction in PDAC [18]. Our study revealed the colocalization of staining for collagen, CD90, and αSMA, which indicates that CD90^+^ PSCs may play an important role in the desmoplastic reaction in PDAC.

### Coexpression of CD90 with CD31

**Figure 8:**
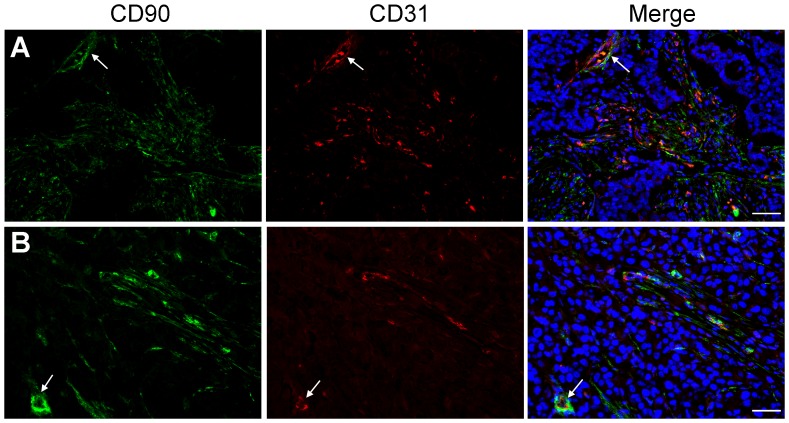
Coexpression of CD90 (*green*) and CD31 (*red*) in PDAC. DAPI counterstaining was used to visualize nuclei (*blue*). Scale bars  = 50 µm. The merged images showed that CD90 was co-localized with CD31 in the tumor vasculature (indicated by arrows) of PDACs.

CD90 expression on the vascular endothelial cells was further confirmed by double fluorescence IHC staining with a vascular endothelial cell marker CD31. As shown in Fig. 8, the merged images showed that, in the tumor vasculature of PDACs, CD90 was co-localized with CD31 (indicated by arrows). CD90 expression on vascular endothelial cells was significantly increased in PDACs of all stages compared to normal pancreas and chronic pancreatitis. The result indicates that CD90 vascular immunostaining could distinguish PDAC from non-malignant pancreatic tissues and the overexpression of CD90 in tumor vasculature of PDAC may play a role in angiogenesis in pancreatic adenocarcinoma. In a previous study, Foygel *et al.* reported that CD90 could serve as a target for PDAC neovasculature imaging due to its significantly increased expression in PDAC vessels [12]. The study only focused on CD90 expression on the vessels and *in vivo* validated CD90 as a promising ultrasound molecular imaging target in PDAC xenografts and transgenic mouse models of PDAC [12]. Our study revealed that the significantly increased expression of CD90 occurred both in PDAC vascular cells and activated PSCs. The double immunofluoresence staining of CD90 (*green*) and CD31 (*red*) on pancreatic neuroendocrine tumor (PNET) showed that CD90 largely overlapped with CD31 (Figure S7 in S1 File), indicating that CD90 might be a promising marker for pancreatic neuroendocrine carcinoma.

### Double immunostaining of CD90 with CD45

Since leukocytes are recruited to the tumor stroma as a consequence of an immune response against tumor, we performed double immunofluorescence (IF) staining of CD90 with CD45 (leukocyte common antigen) to eliminate leukocytes from CD90^+^ stromal cells. As shown in Figure S8 in S1 File, CD45 was abundantly expressed in chronic pancreatitis samples with a limited overlap with CD90 (*yellow*). However, CD45 was negative in early stage PDACs and exhibited sparse expression in late stage PDACs. Interestingly, double staining data showed that CD90^+^ cells were negative for CD45 in 98% (96/98) of PDACs, which excluded leukocytes from the CD90^+^ stromal population. Only 2% (2/98) of PDACs showed a minimum overlap between CD90 and CD45 (Figure S8 in S1 File). CD90 expression on the subpopulation of leukocytes has been reported in HIV-infected patients [19], however, in PDAC, leukocytes were excluded from CD90^+^ stromal cells.

## Discussion

Pancreatic adenocarcinoma (PDAC) is a deadly neoplasm surrounded by a dense reactive stroma called ‘desmoplastic stroma’ [20]. The desmoplastic stroma plays an important role in pancreatic adenocarcinoma initiation, growth and progression and contributes to the aggressiveness of this malignancy by fostering tumor growth and metastatic spread [17], [21]. The stroma of PDAC is composed of different cell types, including fibroblasts, vascular cells, immune cells (leukocytes), and pericytes [17]. Fibroblasts are the most abundant components forming the bulk of PDAC stroma, which are also called cancer-associated fibroblasts (CAFs) or activated pancreatic stellate cells (PSCs) [22], [23]. Numerous studies have demonstrated the importance of interactions between the stroma and malignant epithelial cells of pancreatic adenocarcinoma, confirming that the presence of a desmoplastic stroma is associated with worse clinical outcomes [24].

CD90 is a marker for several types of human stem cells, such as hematopoietic stem cells, hepatic stem/progenitor cells, and mesenchymal stem cells [5], [6]. CD90 is an important regulator of cell-cell and cell-matrix interactions, with significant roles in cellular adhesion and migration, nerve regeneration, and fibrosis [7]. CD90 also plays important roles in oncogenesis and has been identified as a marker for cancer stem cells in various malignancies, such as liver cancer [8], esophageal cancer [9], gastric cancer [25], and glioma [10]. CD90^+^ CSCs not only displayed tumorigenic capacity to initiate tumor and self-renewal, but also conferred an enhanced metastatic potential [8], [9]. Besides the CSC properties of CD90^+^ cells in these cancers, CD90 expression has also been observed in stromal cells (e.g. mesenchymal stem cells, cancer-associated fibroblasts, and endothelium) of various cancers, and plays an important role in disease progression [26], [27], [28].

In the present study, we found that the expression level of CD90 is significantly increased in PDAC and its metastatic cancers compared to that in normal pancreas and non-malignant cases, such as chronic pancreatitis and benign islet tumors. Our immunohistochemical data demonstrated that CD90 was negative in normal pancreas and 82.7% (24/29) of adjacent normal pancreas tissues, with weak and sparse staining restricted on the connective tissues, which are less than 1% CD90^+^ cells. In non-malignant pancreatic disease, such as chronic pancreatitis and benign islet cell tumors, an increased expression of CD90 was observed on the activated fibroblasts. The positive expression of CD90 was observed in 36% (4/11) of chronic pancreatitis and 20% of benign islet cell tumors. Note that no expression of CD90 was observed in pancreatic ducts, acini, islets, or blood vessels in normal pancreas, chronic pancreatitis or benign islet tumor.

In PDACs, CD90 expression was highly overexpressed where 92.8% (91/98) of PDAC specimens were positive for CD90. CD90 was abundantly expressed in stromal cells, including fibroblasts and vascular endothelial cells. Abundant CD90^+^ fibroblasts were present in all stages, which were found closely localized to malignant ducts, as well as blood vessels and pancreatic lobules. Double IF staining of CD90 and CD45 (leukocyte common antigen) showed that CD90^+^ cells were negative for CD45 in 98% (96/98) of PDACs, which excluded leukocytes from the CD90^+^ stromal population. Note that, in stage I, a single layer of CD90^+^ fibroblasts was observed closely localized around duct-like structures. Interestingly, in late stage PDACs, CD90 was also expressed on the apical cell surface of malignant ductal epithelia visualized by immunofluorescence (IF) staining. In addition, moderate to strong CD90 expression was also present in metastatic PDACs to liver, lymph node, abdominal cavity, and omentum. Statistical analysis of IHC score of CD90 expression showed that CD90 was significantly associated with PDAC (*p*<0.0001) and its metastatic cancers (*p*<0.05). The significant overexpression of CD90 in PDAC as compared to normal pancreas and non-malignant cases indicated that CD90 could serve as a candidate marker for pancreatic adenocarcinoma.

The IHC results suggest that CD90, a regulator of cell-cell and cell-matrix interactions [7], may play an important role in tumor-stroma interactions in PDAC and modulation of fibroblast proliferation and migration. The cancer-associated fibroblasts can directly stimulate tumor cell proliferation through provision of various growth factors and cytokines [21]. Tang *et al.* has reported that CD90^+^ cells in esophageal cancer possessed enhanced ability to both invade and migrate [9]. The presence of CD90^+^ cells has been associated with a high incidence of distant organ metastasis in hepatocellular carcinoma [29]. CD90 overexpression was also observed in atypical meningiomas and meningioma metastasis [30]. In this study, double IF staining of CD90 with αSMA revealed that CD90^+^ stromal cells consist largely of activated PSCs in PDAC. Activated PSCs, with characteristics of myofibroblasts [31], exhibit a significant interaction with pancreatic cancer cells that promotes cancer progression [23]. The CD90^+^ stromal cells in PDAC may form an environment that promotes tumor growth as well as regional and distant metastasis [32].

Double fluorescence IHC staining of CD90 and CD24, one of CSC markers for PDAC, showed that there was little overlap between these two markers. However, CD90^+^ cells were found clustered around CD24^+^ malignant ducts, which further suggests that CD90 may be involved in the tumor-stroma interactions and play a role in cancer-associated fibroblasts to promote pancreatic tumorigenesis and development [32]. Double fluorescence IHC staining of CD90 with a vascular endothelial cell marker CD31 demonstrated that CD90 expression on vascular endothelial cells was significantly increased in PDACs of all stages compared to normal and non-malignant pancreatic tissues. The significantly elevated expression of CD90 on vascular endothelial cells in PDAC suggests that CD90 may play a role in tumor angiogenesis where new blood vessels are formed. In addition, signals arising from the CD90^+^ fibroblast-rich stroma may also promote neovascularization [17].

## Conclusions

We have reported herein that CD90 was significantly overexpressed in patients with PDAC as compared to normal pancreas, chronic pancreatitis, and benign islet tumors. The abundant expression of CD90 was mainly localized in the stroma of PDAC of all stages, including cancer-associated fibroblasts (also known as activated pancreatic stellate cells) and vascular endothelial cells. The results suggest that CD90 could serve as a candidate marker for pancreatic adenocarcinoma. The CD90^+^ stromal cells were clustered around malignant pancreatic ductal cells, indicating that CD90 may be involved in the tumor-stroma interaction and establish a favorable environment that promotes tumor progression. To isolate CD90^+^ stromal cells from PDAC by flow cytometry [33] or immunostaining-directed laser capture microdissection (iLCM) [34] could facilitate better understanding of the mechanisms by which CD90^+^ fibrotic stroma promotes tumor growth in pancreatic adenocarcinoma.

## Supporting Information

S1 File Combined Supporting Information file. **Figure S1,** The IHC result of monoclonal IgG Isotype Control on human pancreatic adenocarcinoma tissues by immunoperoxidase and immunofluorescence methods, respectively. **Figure S2,** CD90 expression in the adjacent normal tissue (ANT) of pancreatic adenocarcinoma. **Figure S3,** CD90 expression in benign islet cell tumors. **Figure S4,** Expression of CD90 (*green*) in mtastatic pancreatic adenocarcinoma to liver, lymph node, abdominal cavity, and omentum, respectively. **Figure S5,** Expression of CD90 (*green*) in malignant pancreatic neuroendocrine tumor and adenosquamous carcinoma. **Figure S6,** A representative set of images showing the colocalization of collagen, CD90 (*green*) and αSMA (*red*) staining in stromal area of PDAC. **Figure S7,** Coexpression of CD90 (*green*) and CD31 (*red*) in pancreatic neuroendocrine tumor. **Figure S8,** Double immunofluorescence staining of CD90 (*green*) and CD45 (*red*) in chronic pancreatitis and pancreatic adenocarcinoma of early (I and II) and late (III and IV) stages.
(PDF)
