# Supplementary material for: Overexpression of CD90 (Thy-1) in Pancreatic Adenocarcinoma Present in the Tumor Microenvironment
Source: PLoS One. 2014 Dec 23;9(12):e115507. doi: 10.1371/journal.pone.0115507 (PMC4275230; doi:10.1371/journal.pone.0115507)
Supplement: S1 File — Combined Supporting Information file. Figure S1, The IHC result of monoclonal IgG Isotype Control on human pancreatic adenocarcinoma tissues by immunoperoxidase and immunofluorescence methods, respectively. Figure S2, CD90 expression in the adjacent normal tissue (ANT) of pancreatic adenocarcinoma. Figure S3, CD90 expression in benign islet cell tumors. Figure S4, Expression of CD90 (green) in mtastatic pancreatic adenocarcinoma to liver, lymph node, abdominal cavity, and omentum, respectively. Figure S5, Expression of CD90 (green) in malignant pancreatic neuroendocrine tumor and adenosquamous carcinoma. Figure S6, A representative set of images showing the colocalization of collagen, CD90 (green) and αSMA (red) staining in stromal area of PDAC. Figure S7, Coexpression of CD90 (green) and CD31 (red) in pancreatic neuroendocrine tumor. Figure S8, Double immunofluorescence staining of CD90 (green) and CD45 (red) in chronic pancreatitis and pancreatic adenocarcinoma of early (I and II) and late (III and IV) stages. (PDF) [file pone.0115507.s001.pdf]

File S1. Combined Supporting Information file.

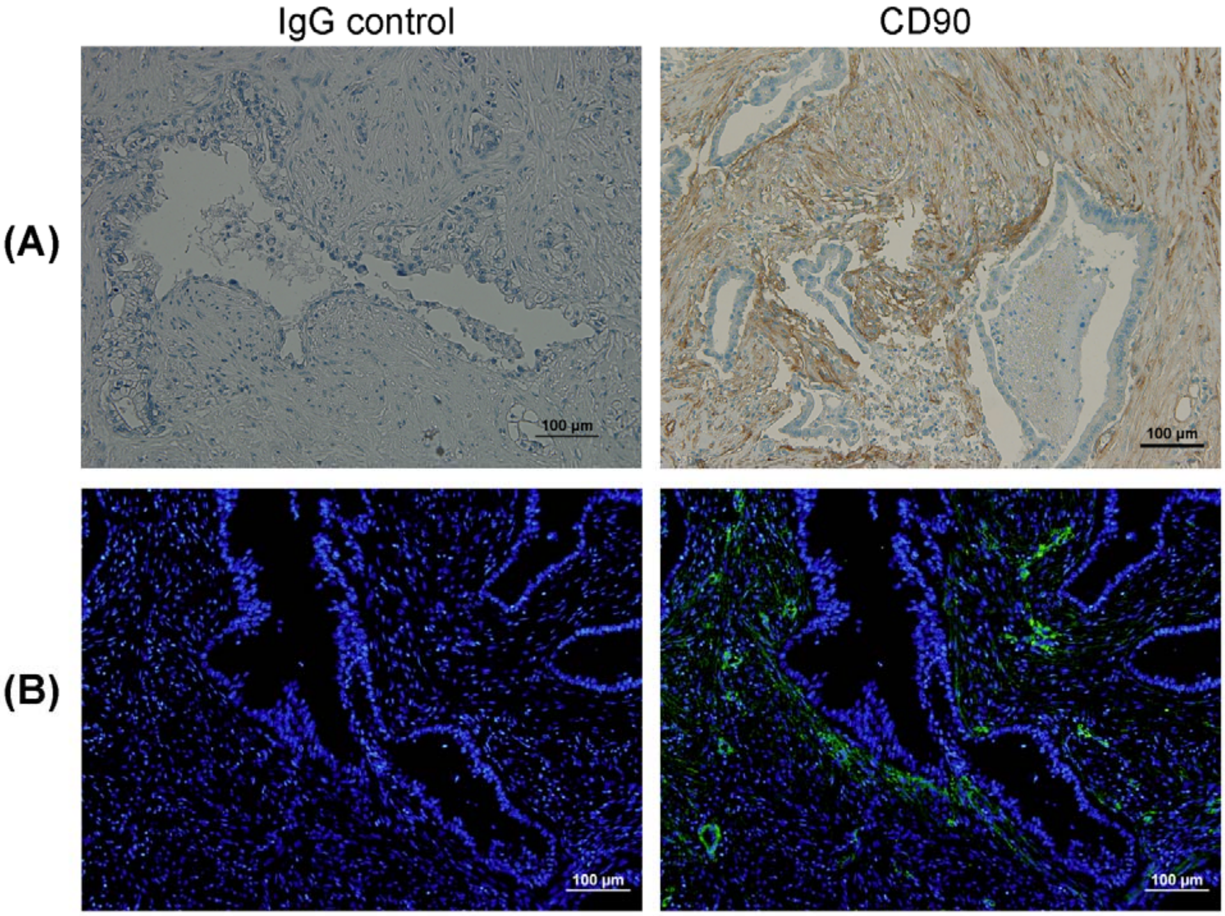

Figure S1. Immunohistochemistry on human pancreatic adenocarcinoma tissues stained with 10 µg/mL rabbit anti-human monoclonal IgG Isotype Control (left panel) and 10 µg/mL rabbit anti-human monoclonal CD90 antibody (right panel) by immunoperoxidase (A) and immunofluorescence (B) methods. (A) CD90 staining was shown in *brown*. Nuclei were counterstained with hematoxylin. (B) CD90 staining was shown in *green*. Nuclei were stained with DAPI (*blue*). Scale bars = 100 µm.

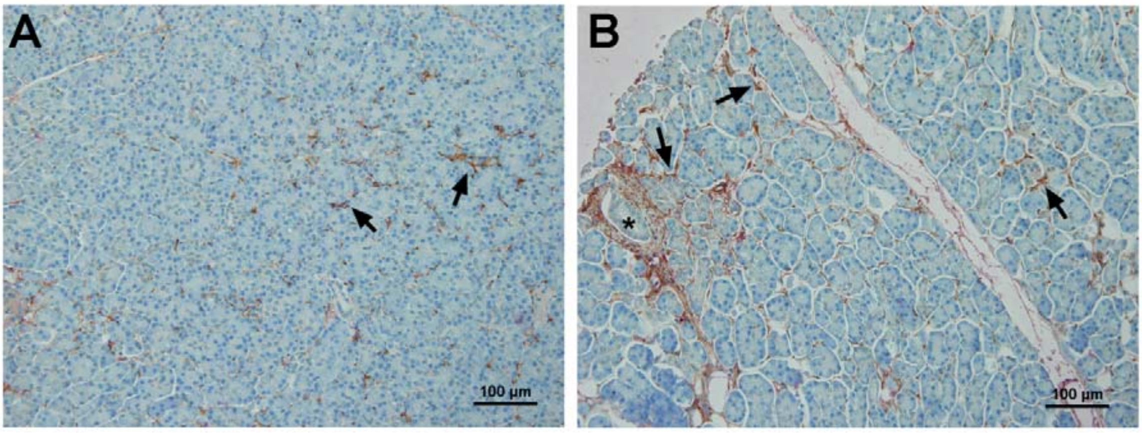

Figure S2. CD90 expression in the adjacent normal tissue (ANT) of pancreatic adenocarcinoma. (A) A representative image of ANT shows minimal expression of CD90 on the connective tissues (indicated by arrow). (B) The fibroblasts around the pancreatic duct (asterisk) in ANT showed increased CD90 expression compared to those in normal pancreas. No expression of CD90 was observed on acini, islets, and ductal epithelia. Scale bars = 100 µm.

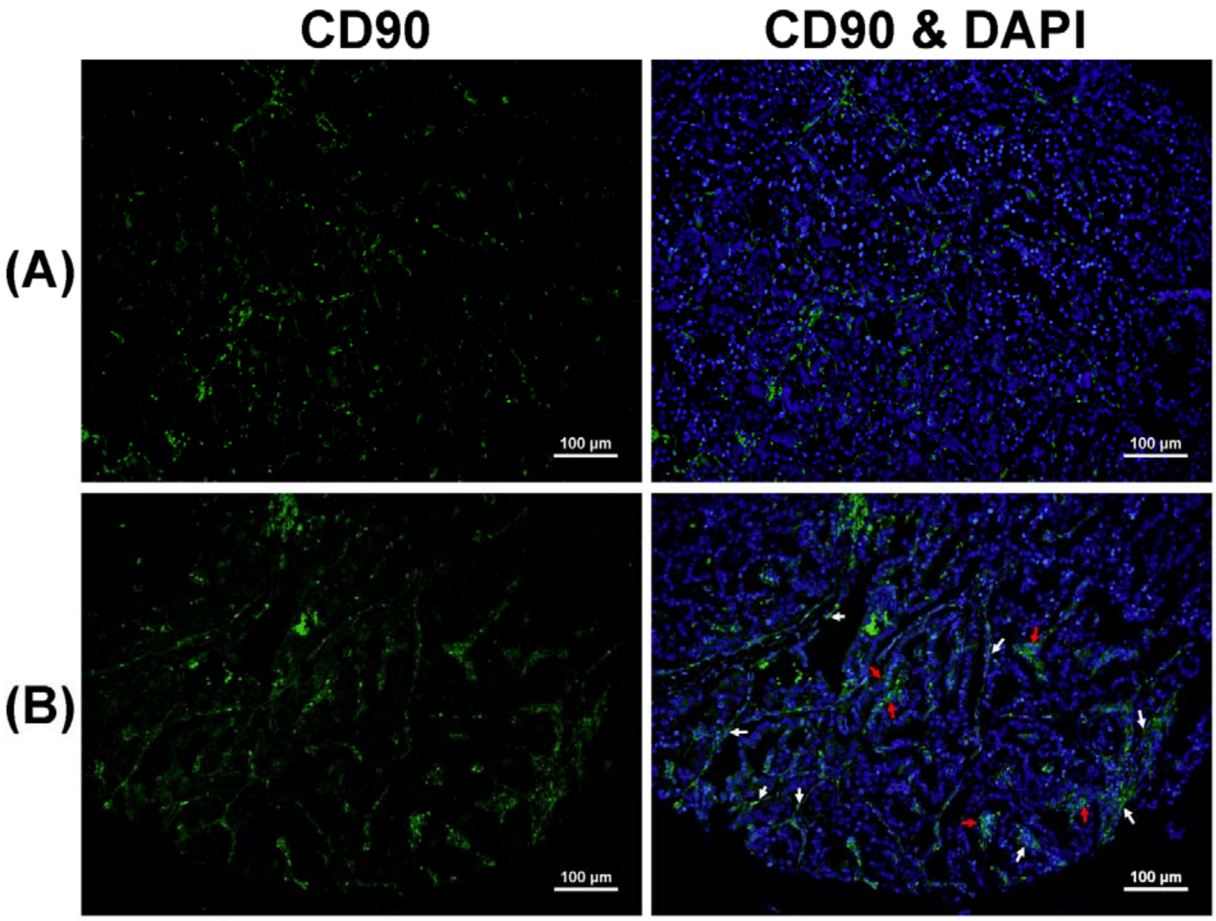

Figure S3. CD90 expression in benign islet cell tumors. (A) An islet cell tumor showed moderate CD90 expression in fibroblast cells. (B) A representative image of benign islet tumor shows strong CD90 expression in both fibroblasts (white arrow) and tumor cells (red arrow). Nuclei were stained with DAPI (*blue*). Scale bars = 100 µm.

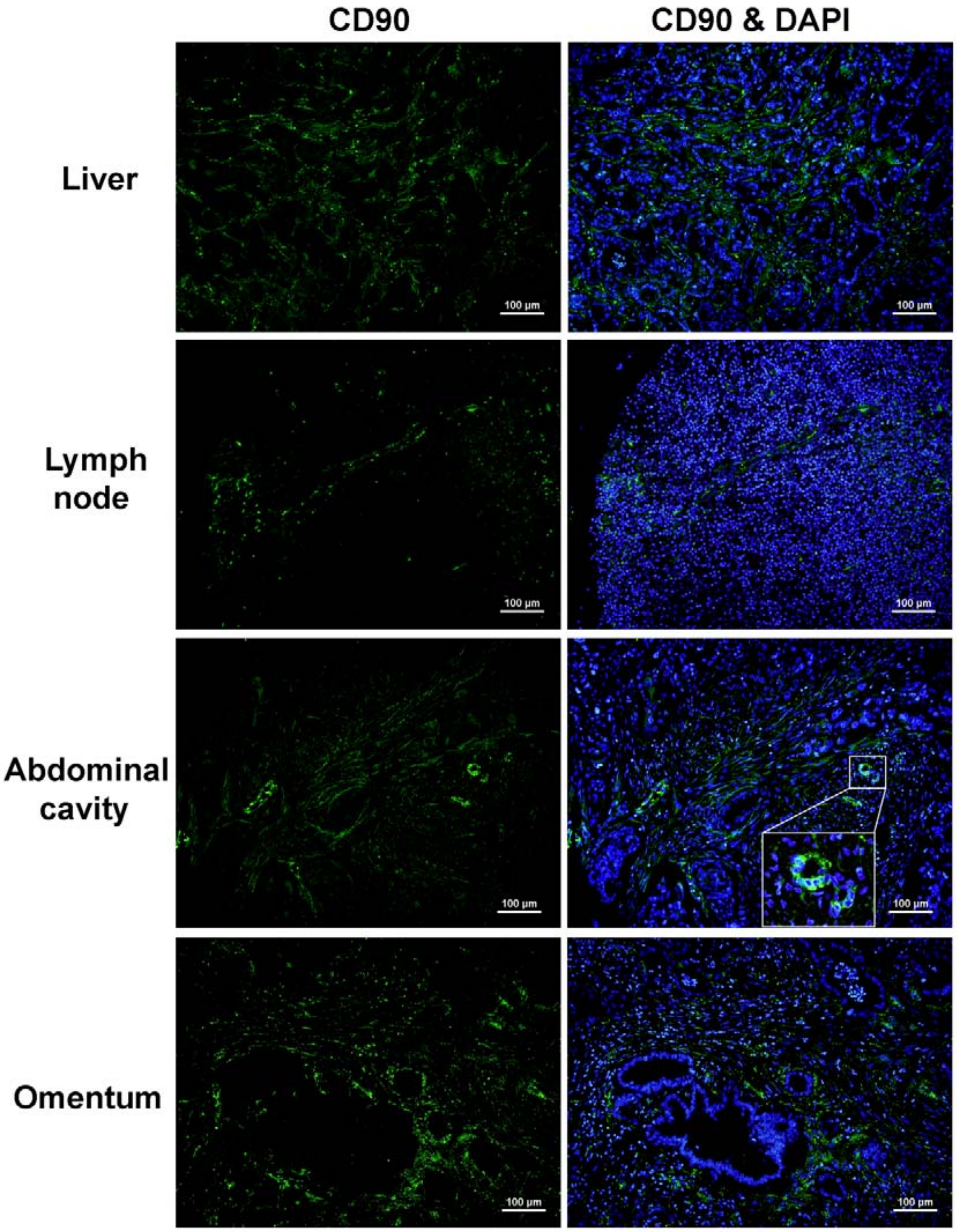

Figure S4. Expression of CD90 (*green*) in metastatic pancreatic adenocarcinoma that has spread to liver, lymph node, abdominal cavity, and omentum, respectively. DAPI counterstaining was used to visualize nuclei (*blue*). Strong CD90 expression was observed on fibroblasts in metastatic carcinomas to liver, lymph node, abdominal cavity, and omentum. The insert showed expression of CD90 on the vascular niche in abdominal cavity. Scale bars = 100 µm.

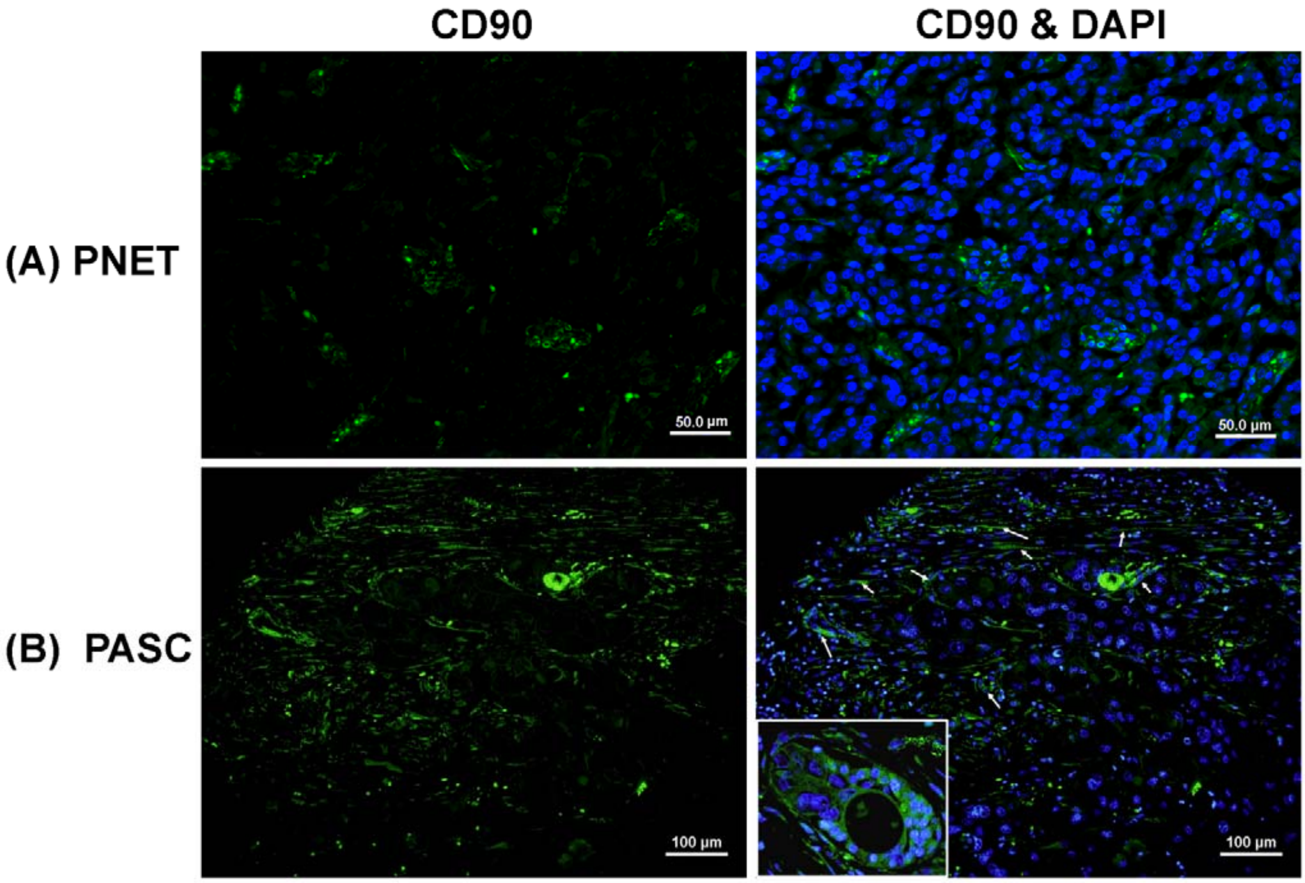

Figure S5. Expression of CD90 (*green*) in (A) malignant pancreatic neuroendocrine tumor (PNET) and (B) pancreatic adenosquamous carcinoma (PASC). CD90 expression (*green*) was present on tumor cells in PNET. However, strong CD90 expression was observed in both stromal cells (arrows) and tumor cells. The insert in B is a higher magnification showing CD90 expression on tumor cells in PASC.

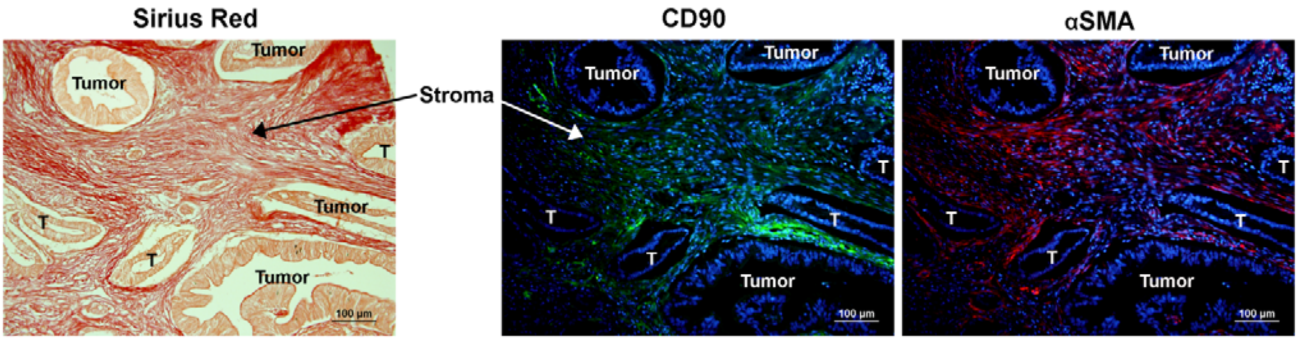

Figure S6. A representative set of images showing the colocalization of collagen, CD90 (*green*) and αSMA (*red*) staining in stromal area of PDAC. The stroma exhibits strong positive staining for collagen as well as CD90 and αSMA, indicating the presence of $CD90^+$ activated PSCs in the desmoplastic reaction in PDAC. (T: Tumor)

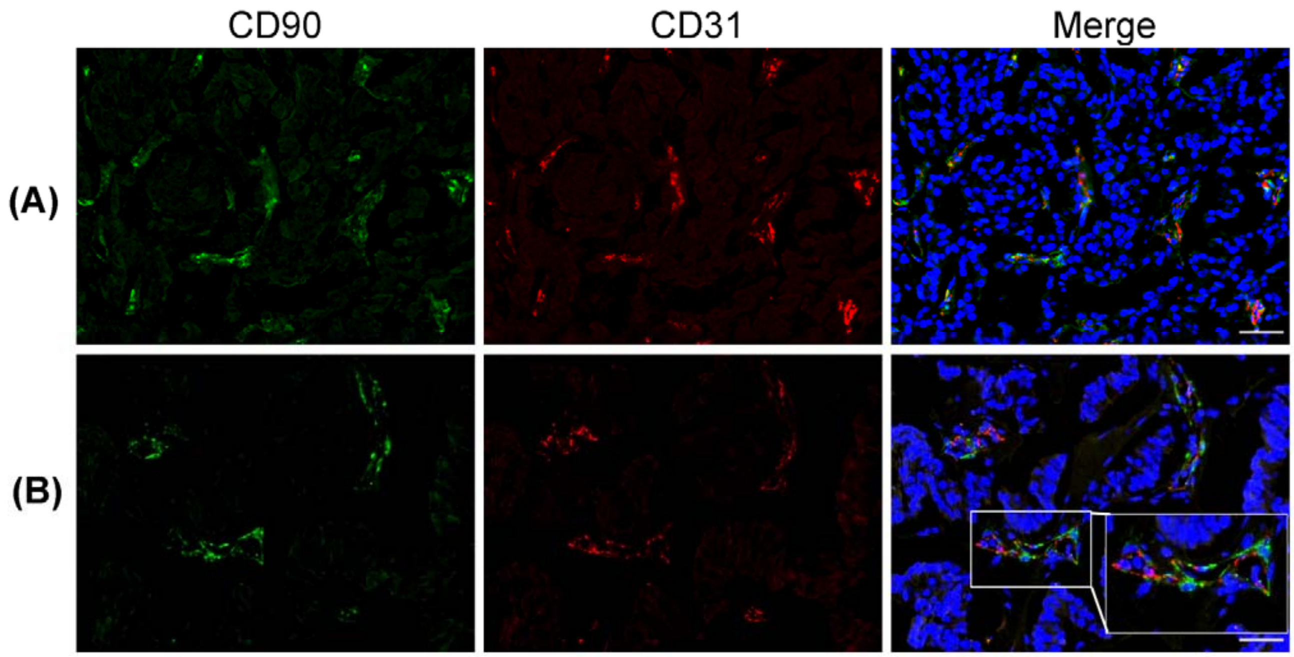

Figure S7. Coexpression of CD90 (*green*) and CD31 (*red*) in malignant pancreatic neuroendocrine tumor (PNET) (A: well differentiated; B: moderately differentiated). DAPI counterstaining was used to visualize nuclei (*blue*). CD90 and CD31 were largely overlapped (*yellow* or *orange*) as shown in the merged images. The insert in B is a higher magnification showing the overlap between CD90 and CD31. Scale bars = 50 μm.

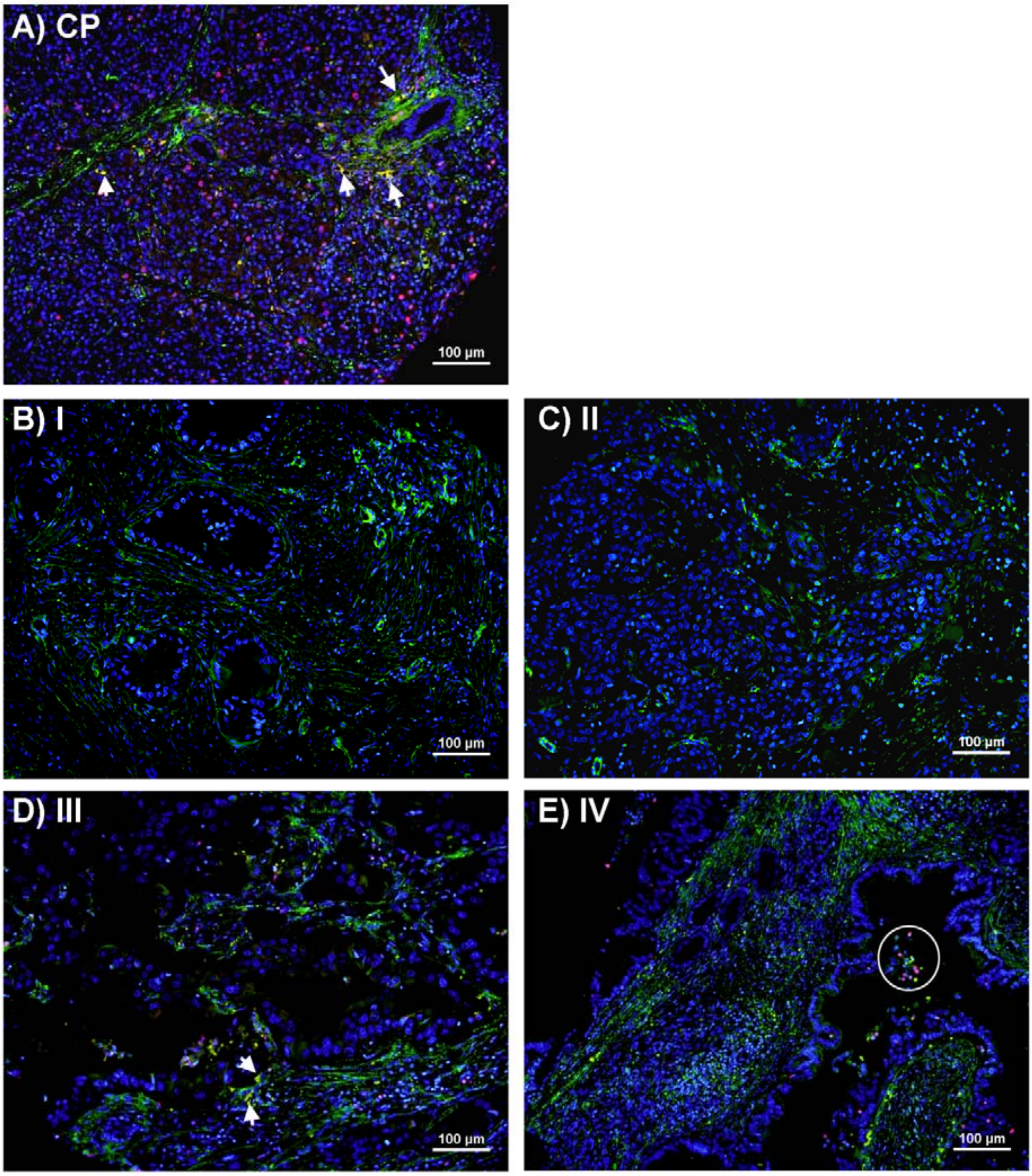

Figure S8. Double immunofluorescence staining of CD90 (*green*) and CD45 (*red*) in chronic pancreatitis (CP) and pancreatic adenocarcinoma of early (I and II) and late (III and IV) stages. DAPI counterstaining was used to visualize nuclei (*blue*). (A) A pancreatitis tissue specimen showed abundant expression of CD45 (*red*) and a limited overlap (*yellow*) with CD90 (indicated by arrow). (B and C) Early stage PDACs showed abundant expression of CD90 (*green*) in stroma, but were negative for CD45. Late stage PDACs (D and E) showed abundant expression of CD90 (*green*) and sparse

expression of CD45 (*red*). (D) A PDAC specimen at stage III showed a minimum overlap (*yellow*) between CD90 and CD45 (indicated by arrow), which was observed in less than 0.5% $CD90^+$ cells. (E) A PDAC specimen at stage IV showed sparse expression of CD45 (highlighted with a white circle), but there was no overlap between CD90 and CD45. Scale bars = 100 μm.
